# A publicly available deep learning model and dataset for segmentation of breast, fibroglandular tissue, and vessels in breast MRI

**DOI:** 10.1038/s41598-024-54048-2

**Published:** 2024-03-05

**Authors:** Christopher O. Lew, Majid Harouni, Ella R. Kirksey, Elianne J. Kang, Haoyu Dong, Hanxue Gu, Lars J. Grimm, Ruth Walsh, Dorothy A. Lowell, Maciej A. Mazurowski

**Affiliations:** Department of Radiology, Duke University Medical Center, Box 2731, Durham, NC 27710 USA

**Keywords:** Breast cancer, Risk factors, Data publication and archiving, Predictive medicine

## Abstract

Breast density, or the amount of fibroglandular tissue (FGT) relative to the overall breast volume, increases the risk of developing breast cancer. Although previous studies have utilized deep learning to assess breast density, the limited public availability of data and quantitative tools hinders the development of better assessment tools. Our objective was to (1) create and share a large dataset of pixel-wise annotations according to well-defined criteria, and (2) develop, evaluate, and share an automated segmentation method for breast, FGT, and blood vessels using convolutional neural networks. We used the Duke Breast Cancer MRI dataset to randomly select 100 MRI studies and manually annotated the breast, FGT, and blood vessels for each study. Model performance was evaluated using the Dice similarity coefficient (DSC). The model achieved DSC values of 0.92 for breast, 0.86 for FGT, and 0.65 for blood vessels on the test set. The correlation between our model’s predicted breast density and the manually generated masks was 0.95. The correlation between the predicted breast density and qualitative radiologist assessment was 0.75. Our automated models can accurately segment breast, FGT, and blood vessels using pre-contrast breast MRI data. The data and the models were made publicly available.

## Introduction

Algorithmic quantitative analysis of medical images could lead to improved diagnostic accuracy and prognosis for patients, but this is hindered by multiple factors. These include: (1) poor availability of shared data that could be used for the development of algorithmic models and as a common benchmark for model evaluation, (2) poor public availability of the developed models that would allow for comparison between different approaches and use for downstream tasks, and (3) imprecise definitions of the quantities measured in images, leading to inconsistent model development and evaluation with high inter-reader variability when these quantities are assessed by human readers, such as radiologists. An example of a measurement that suffers from all these drawbacks is breast density.

Breast density has strong clinical implications despite its qualitative assessment methods. Many studies have shown that women with a higher breast density are at a greater risk for developing breast cancer^1–4^. Breast density, or the amount of fibroglandular tissue (FGT) relative to overall breast volume, is typically assessed by a radiologist qualitatively or by using semi-automatic quantitative tools. However, both methods still have inter-user variability, as radiologists use qualitative descriptors to perform categorization (e.g. “scattered” vs. “heterogenous” vs. “extreme”) with kappa values ranging from 0.69 to 0.84 for inter-radiologist agreement, and some semi-automatic tools have been shown to underestimate breast density^5–9^. Therefore, there is a need for an objective and efficient method of automated breast density assessment.

The imaging modality that allows for the highest contrast between tissues within the breast is magnetic resonance imaging (MRI). It is used to screen high-risk women and stage women with newly diagnosed breast cancer, among other indications^10^. It has many advantages over standard mammography, including increased sensitivity, three-dimensional visualization of the breast and axilla, and dynamic information about blood flow^11^. Due to the nature of MRI usage in high-risk populations and the importance of accurate risk assessment models, an accurate assessment of FGT is needed.

Previous studies have utilized deep learning to perform automated segmentation of FGT on breast MRI but with significant drawbacks, mainly regarding private dataset usage^12–16^. The lack of annotated large-scale datasets has been widely recognized as one of the most significant challenges in medical imaging segmentation^17,18^. Previous works also do not clearly define the annotation methods used and there can be ambiguity when defining the boundaries of tissues, especially the breast which has limited surrounding anatomical landmarks. Providing full transparency facilitates the reproduction of results and allows others to further improve on the achieved results. Furthermore, to our knowledge, previous studies do not take blood vessels into account when performing manual segmentations. Even in pre-contrast MRI sequences, large blood vessels, namely branches of the internal mammary and lateral thoracic arteries, can still appear hyperintense and therefore be confused with FGT. The initial branches of these larger arteries can occupy a significant amount of volume within the breast, which can confound FGT calculations.

In our study, we address prior limitations to improve the assessment of FGT on breast MRI with our publicly available dataset and segmentation method. The contributions of the study are as follows: (1) We provide a detailed and clear methodology to annotate breast MRI that allows for a higher level of reproducibility with a clearer, quantitative definition of breast density. In addition to being explicit, our methodology is the first to clearly consider vessels as separate structures. (2) We generated an extensive set of detailed, pixel-wise, fully three-dimensional annotations, a result of hundreds of hours spent annotating by our team. The average annotation time per case is 8 hours. The annotations were all modified and/or approved by breast fellowship-trained radiologists. (3) The annotations are publicly available at The Cancer Imaging Archive website along with the corresponding MRIs previously made available by our group. (4) We developed and evaluated an accurate deep learning model to segment the breast, FGT, and blood vessels. (5) Finally, we made the segmentation model publicly available so that others can apply it to their own datasets.

## Methods

### Dataset

We randomly selected 100 cases from the publicly available Duke-Breast-Cancer-MRI dataset which is available at 10.7937/TCIA.e3sv-re93^19^. The full dataset contains MRI sequences from 922 biopsy-confirmed invasive breast cancer patients at the Duke University Medical Center.

Our study was determined to be exempt from review by the Duke Health Institutional Review Board. The review board also determined that receiving informed consent is waived as we are using a publicly available database. All methods were performed accordance with the relevant guidelines and regulations.

The MRIs were obtained from patients in the prone position using a 1.5 T or 3.0 T scanner (GE Healthcare and Siemens). Axial images were acquired with a voxel size ranging from 320 × 320 × 144 to 512 × 512 × 200 and a resolution ranging from 0.6 × 0.6 × 1.0 mm^3^ to 1.1 × 1.1 × 1.2 mm^3^. We used the T1-weighted fat-suppressed pre-contrast sequence for our study as it provides ample contrast between FGT and fatty tissue while minimizing visible blood vessels.

The 100 patients were randomly split into training, validation, and test sets with a 70%/15%/15% split. The validation set was used during model development and evaluation to improve model performance. The test set was used only after model development was completed and no changes were made to improve performance on the test set data after its use for evaluation.

### Image annotations

All MRIs were manually annotated and reviewed using 3D Slicer. There were three target tissues for annotation: breast, FGT, and blood vessels. The annotators and reviewers were given the following instructions:

1. Breast annotation: Trace the contours of the breast, excluding inner anatomical structures, such as the chest wall muscles and sternum. The superior edge of the annotation should stop approximately at the level of the clavicle. The inferior edge of the annotation should stop at the inframammary fold.
2. FGT annotation: Mark all areas of FGT that do not appear to be blood vessels. Biopsy clips and lymph nodes should be excluded. All FGT should be within the breast.
3. Blood vessel annotation: Mark all readily apparent blood vessels. All blood vessels should be within the breast.

The annotators consisted of one postdoctoral fellow, one upper-level medical student, and two undergraduate students who were all given the same annotation instructions, examples, and instructions to use the software. All annotators had a minimum of 5 hours spent learning how to properly perform annotations and reviewing practice annotations to ensure that instructions were followed properly. The medical student annotator reviewed and, if needed, edited all annotations. All annotations were then reviewed and, if needed, edited by one of three board-certified, fellowship-trained breast radiologists at our institution.

The annotations are publicly available online at 10.7937/TCIA.e3sv-re93, under “Data Access”, “Supplemental Segmentation”, “3D Breast and FGT MRI Segmentation Supplemental Data”. There are additional segmentations included in the database, but this study exclusively used the more extensive “3D Breast and FGT MRI Segmentation Supplemental Data” that were created using the guidelines described above.

### Data processing and model architecture

Multiple steps were taken to improve data uniformity. First, spatial information from DICOM files was used to ensure all MRI volumes and their accompanying annotations were in the same position. Volumes or annotations that were rotated and/or flipped incorrectly were aligned accordingly. Following this, we performed basic preprocessing of the data. First, we capped the extreme values in the image (below 0.1 percentile and above 99.9 percentile) and normalized the intensity values using a z-score, as shown in Eq. (1).

1 $$Z= \frac{x-\mu }{\sigma }$$

Where $x$ is the intensity value of the individual voxel, $\mu$ is the mean intensity value within the MRI volume, and $\sigma$ is the standard deviation for intensity values within the MRI volume.

We selected the U-Net architecture as it has been successfully applied to many medical imaging segmentation tasks^20–22^. The 3D U-Net architecture features a fully connected convolutional residual network with a contraction and expansion phase. During the contraction phase, each step applies two 3 × 3 × 3 convolutional layers with batch normalization and a rectified linear unit (ReLU) followed by a 2 × 2 × 2 max pooling layer with a stride 2. During the expansion phase, each step up-samples the input, concatenates it with its corresponding feature map from the contracting path, then applies two 3 × 3 × 3 convolutional layers with batch normalization and a ReLU. We also used a 2D U-Net that featured a similar architecture with one less dimension used in all layers.

**Figure 1:**
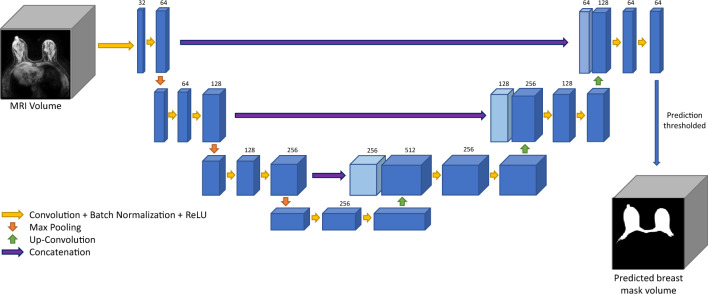
Overview of the data input and U-Net model used to perform breast segmentations.

**Figure 2:**
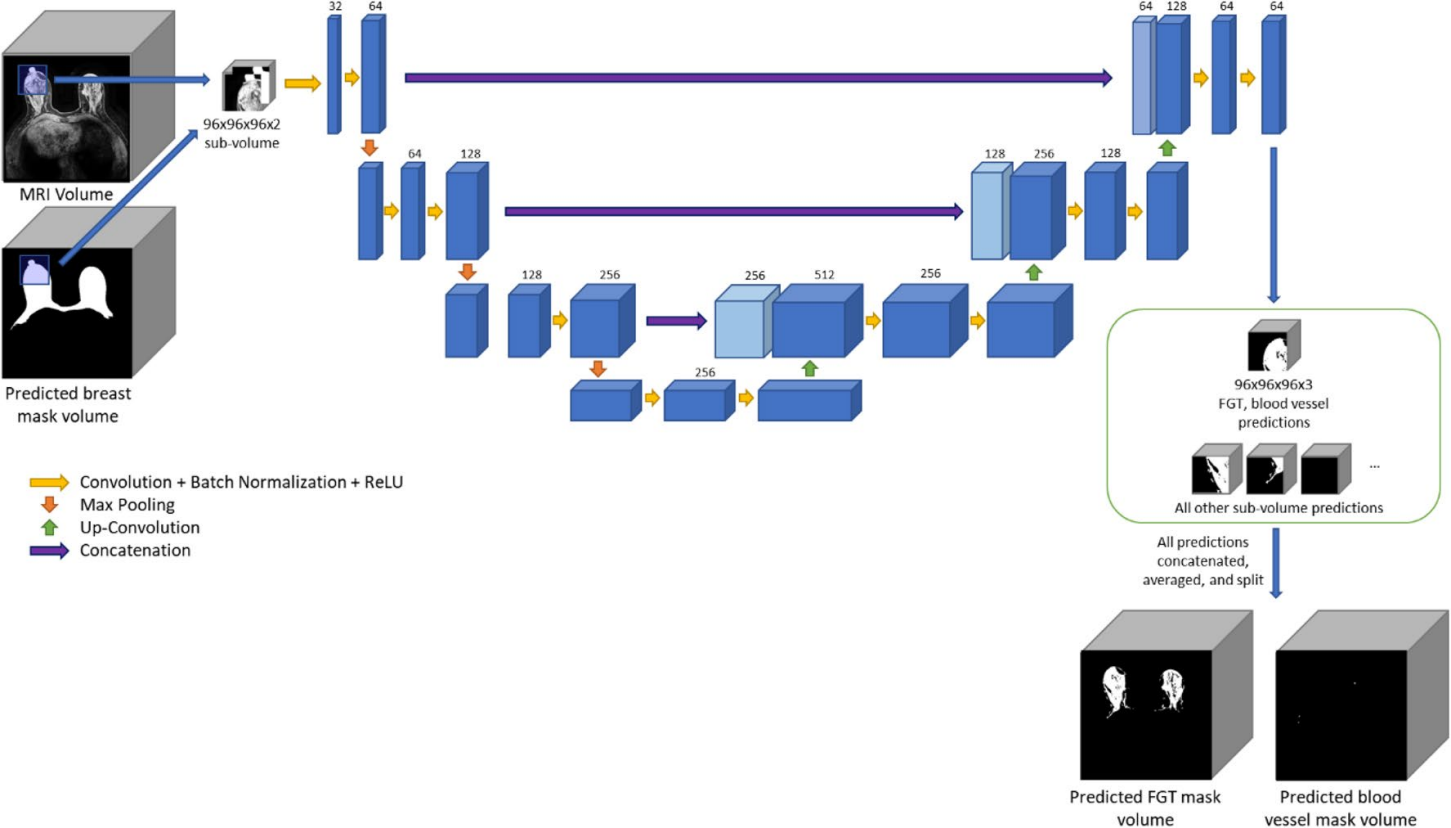
Overview of the data input and U-Net model used to perform FGT and blood vessel segmentations.

Two models were trained for our segmentation task. The first model, named Breast U-Net, used the MRI volume to output a binary prediction on each voxel to perform a segmentation of the breast. For the second model, named FGT-Vessel U-Net, we incorporated information on the predicted location of the FGT and vessels, since they will always be contained within the breast. To accomplish this, we included the outputs of Breast U-Net as an additional channel alongside the MRI volume. An overview of the model architecture and data input is shown in Figs. 1 and 2.

We tested two segmentation methods: one with 2D inputs and one with 3D inputs. For 2D inputs, each slice of the volume was inputted into the model individually. For 3D inputs, we developed different methods of inputting the data into the models. To predict the breast, the full MRI volume was input into the model after downsizing to 144 × 144 × 96. To predict the FGT and blood vessels, we divided the 3D MRI volume into 3D sub-volumes of size 96 × 96 × 96. To obtain each sub-volume, we used a random sub-sampling method, where all areas of the volume had an equal chance to be sampled, to generate sub-volumes for model input. Data augmentations that added random motion, noise, and bias fields were used in both models. To obtain segmentations for a full volume, 192 evenly spaced sub-volumes are predicted from the full volume. The prediction for a voxel in the full volume was the mean of all predictions in sub-volumes that contain it. These different 3D approaches for breast and FGT were selected based on experimental results using the training and validation data.

To select hyperparameters, we experimentally evaluated various total epochs, learning rates, learning rate decays, batch sizes, sub-volume sizes, and sub-volume counts using the validation set. Total epochs, learning rate, and learning rate decay were adjusted with the aim of reducing overfitting. We also compared using cross-entropy and dice loss functions. Dice loss in particular was used as blood vessels occupy a small number of voxels and therefore face a class imbalance issue, which can be overcome using dice loss or similar loss functions^23,24^. We chose the model that had the best DSC values for each task after testing various hyperparameter combinations using the validation set. We used the Adam optimizer during gradient back-propagation.

All code and trained models used in the study are publicly available at: https://github.com/mazurowski-lab/3D-Breast-FGT-and-Blood-Vessel-Segmentation.

### Model evaluation

The models were evaluated using the Dice Similar Coefficient (DSC) as it is commonly used to evaluate the performance of image segmentation methods. The minimum DSC value of 0 indicates no overlap between segmentations and the maximum DSC value of 1 indicates complete overlap. For breast segmentation predictions, we applied a sigmoid function to model outputs and thresholded values at 0.5 to generate breast masks. For FGT and blood vessel segmentation predictions, we applied a softmax function to model outputs and assigned each voxel the class with the highest value (FGT, blood vessel, or neither). DSC scores were then calculated for each predicted segmentation individually compared to their respective annotations. The final model evaluation was performed using the test set. The Mann-Whitney test was utilized to compare DSC scores of the test set data.

We used the Pytorch open-source machine learning framework. Our program was run on multiple GPUs (NVIDIA RTX 3090 24GB).

### Additional analysis

Three fellowship-trained and board-certified radiologists were asked to assess the breast density of each image in the test set. Breast density is defined in the Breast Imaging-Reporting and Data System (BI-RADS) Atlas as four categories: (a) almost entirely fat, (b) scattered FGT, (c) heterogeneous FGT, and (d) extreme FGT^25^. We compared assessments by each pair of radiologists using Cohen’s kappa.

FGT percentage was used to assess the model’s predictions in relation to breast density assessments and is calculated using Eq. (2). We used Pearson’s correlation coefficient to compare FGT percentages and radiologist breast density assessments.

2 $$FGT \; Percentage= \frac{Number \; of \; voxels \; labeled \; as \; FGT}{Numer \; of \; voels \; labeled \; as \; breast}$$

## Results

An overview of the demographics and basic characteristics of the 100 patients used in model development and testing is shown in Table 1.

**Table 1:** Patient demographics and tumor staging.

Characteristic	Patients (n = 100)
Age (years)	52.1 ± 10.9
Race (N)	
White	74 (74%)
African American	19 (19%)
Asian	2 (2%)
Hispanic	2 (2%)
American Indian	1 (1%)
Native American	1 (1%)
Multi-racial	1 (1%)
Menopausal status	
Positive	50 (50%)
Negative	49 (49%)
Unknown	1 (1%)
Staging	
Tumor size (N)	
T1	47 (47%)
T2	41 (41%)
T3	9 (9%)
T4	3 (3%)
Regional lymph nodes (N)	
N0	51 (51%)
N1	34 (34%)
N2	6 (6%)
N3	5 (5%)
Unknown	4 (4%)
Metastasis (N)	
M0	76 (76%)
M1	5 (5%)
MX	19 (19%)
Unknown	10 (10%)

The average DSC values for breast, FGT, and blood vessel segmentation can be found in Table 2. The following describes model performance on the test set. The 3D Breast U-Net achieved a DSC score of 0.92 for breast. The following 3D FGT-Vessel U-Net that combined MRI data with predicted breast masks achieved a DSC score of 0.86 for FGT and 0.65 for blood vessels. The 3D FGT-Vessel U-Net that utilized MRI input alone achieved a DSC score of 0.86 for FGT and 0.61 for blood vessels. Between these two 3D FGT-Vessel U-Net, there was no difference between the performance of FGT (*p *= 0.53) or blood vessel (*p* = 0.25) segmentation. The 2D Breast U-Net found a DSC score of 0.95 for breast which was statistically different from the 3D performance (*p* ≤ 0.001). The following 2D FGT-Vessel U-Net that combined MRI data with predicted breast masks achieved a DSC score of 0.84 for FGT and 0.53 for blood vessels. The same model with MRI input alone found a DSC score of 0.84 for FGT and 0.37 for blood vessels. The 2D FGT-Vessel U-Net version had no difference between FGT segmentation performance (*p* = 0.53) but a difference was present between blood vessel predictions (*p* = 0.001). Comparing 3D and 2D FGT-Vessel U-Nets that utilized both predicted breast masks and MRI data input, there was no difference between FGT segmentation performance (*p* = 0.18) but a difference was present between blood vessel performance (*p* < 0.001).

**Table 2:** DSC values for different methods used on test set data.

Method	Breast (DSC value)	FGT (DSC value)	Blood Vessels (DSC value)
2D Input w/breast mask	0.95	0.84	0.37
2D Input alone	0.95	0.84	0.53
3D Input w/breast mask	0.92	0.86	0.65
3D Input alone	0.92	0.86	0.61

DSC, Dice Similar Coefficient; FGT, fibroglandular tissue.

**Figure 3:**
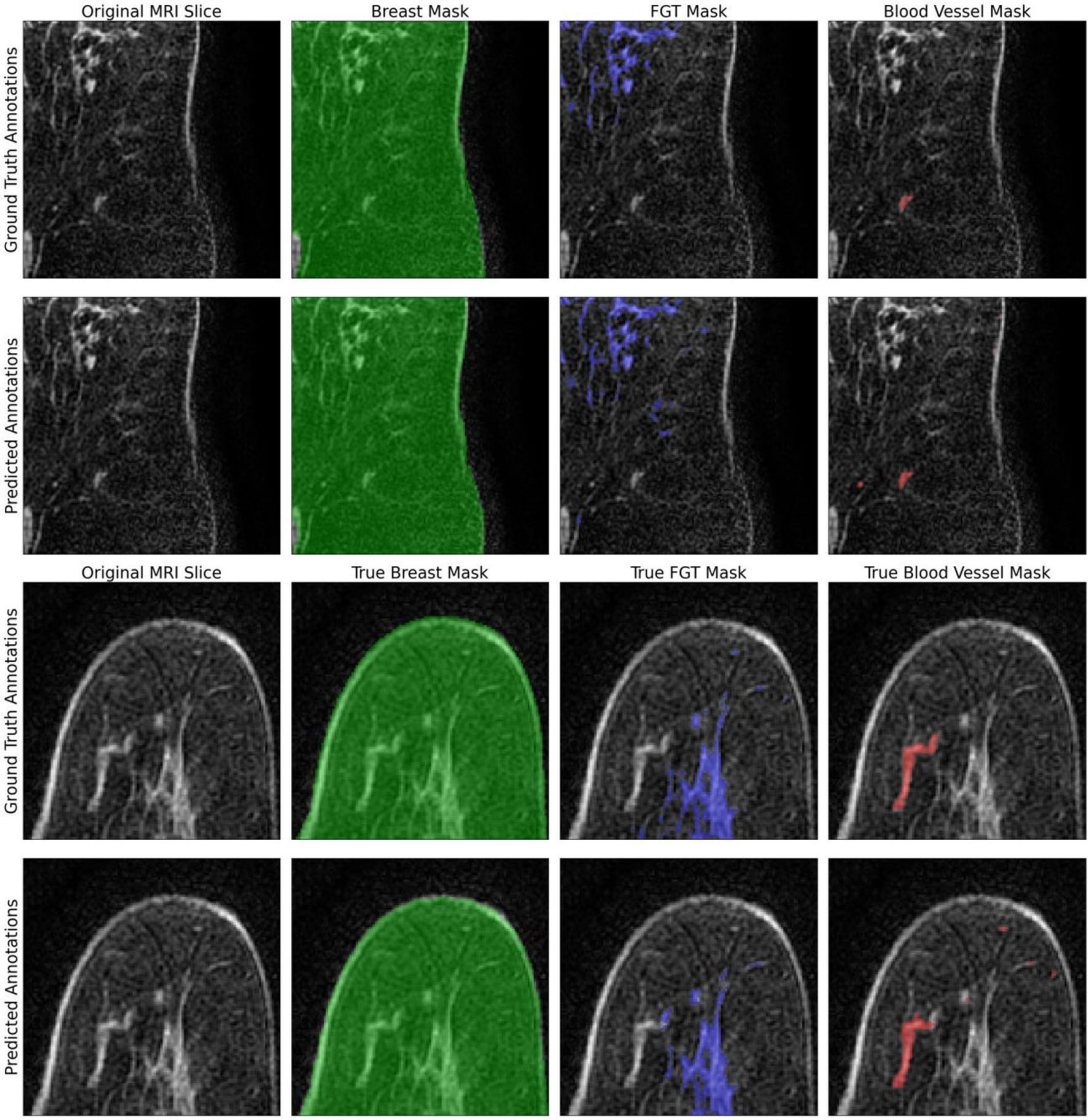
Examples of true and predicted segmentations for breast, FGT, and blood vessels on cropped images from the test set. Breast masks are colored green, FGT masks are colored blue, and blood vessel masks are colored red.

On average, completing the segmentation of a case took 9.6s for the 3D Breast U-Net and 72.3s for the 3D FGT-Vessel U-Net. Sample outputs, taken from the validation set, of the best performing model are shown in Fig. 3.

Table 3 demonstrates the confusion matrices for the breast density assessments of each pair of the 3 radiologists on the test set images. The Cohen’s kappa coefficient was 0.38 for radiologists 1 and 2, 0.65 for radiologists 1 and 3, and 0.43 for radiologists 2 and 3. The cumulative breast density assessments by the 3 radiologists on the 15 test set images were the following: 1 (2.2%) almost entirely fat, 25 (55.6%) scattered FGT, 14 (31.1%) heterogeneous FGT, and 5 (11.1%) extreme FGT.

**Table 3:** Confusion matrices of breast density assessments on test set images between Radiologist 1, 2, and 3.

κ=0.38		R1			
		a	b	c	d
R2	a	0	0	0	0
	b	1	6	0	0
	c	0	2	3	3
	d	0	0	0	0

κ=0.65		R1			
		a	b	c	d
R3	a	0	0	0	0
	b	1	8	1	0
	c	0	0	2	1
	d	0	0	0	2

κ=0.43		R2			
		a	b	c	d
R3	a	0	0	0	0
	b	1	8	1	0
	c	0	0	2	1
	d	0	0	0	2

(a) almost entirely fat, (b) scattered FGT, (c) heterogeneous FGT; (d) extreme FGT. R1, radiologist 1; R2, radiologist 2; R3, radiologist 3.

**Figure 4:**
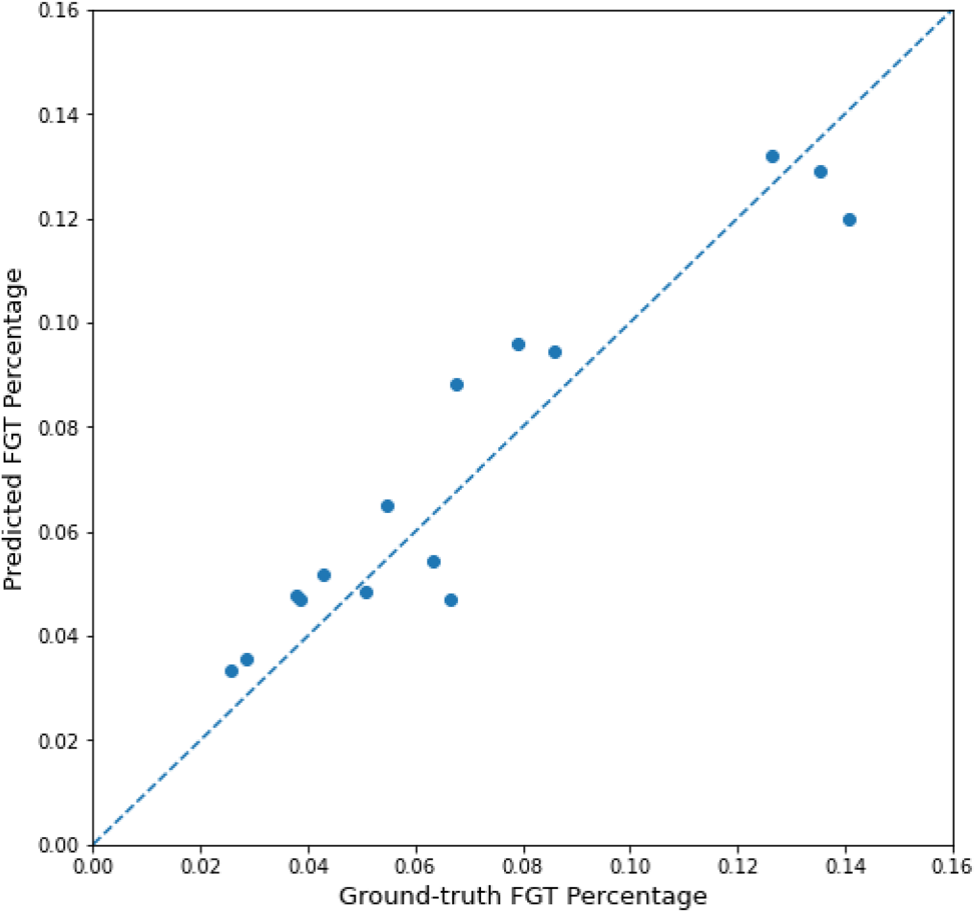
Scatter plot of ground truth FGT percentages versus predicted FGT percentages for images in the test set. The dashed line represents perfect correlation between the two FGT percentages. The average difference between ground-truth and predicted FGT percentage was 1.1% and the Pearson coefficient was 0.95.

Figure 4 compares ground-truth FGT percentages with predicted FGT percentages in the test set. The average difference between the ground-truth FGT percentage and model-predicted FGT percentage was 1.1% and the Pearson coefficient comparing these percentages was 0.95.

**Figure 5:**
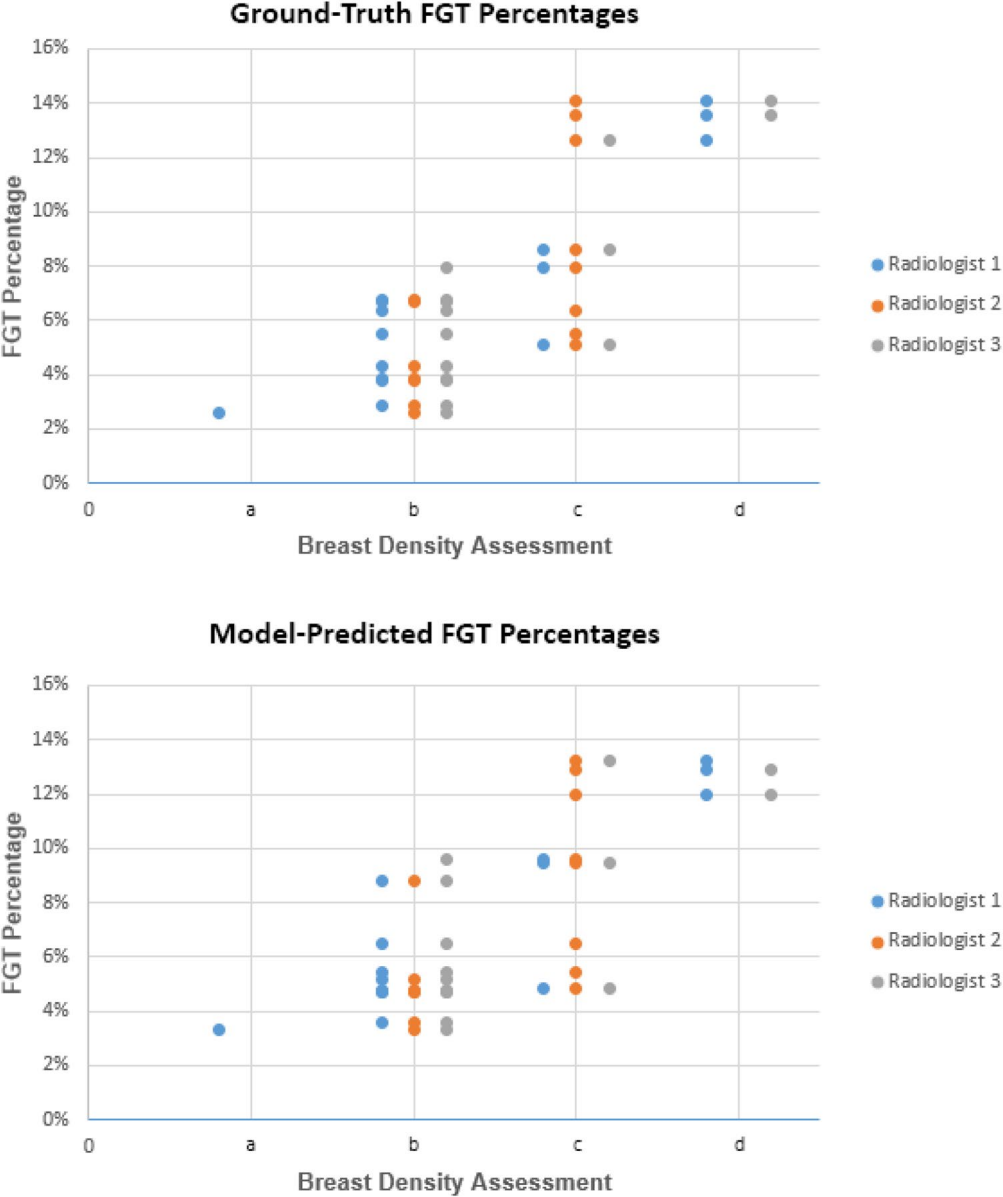
Scatter plot of breast density assessments of 3 radiologists versus FGT percentage of ground-truth annotations and model predictions of test set data. (**a**) almost entirely fat, (**b**) scattered FGT, (**c**) heterogeneous FGT; (**d**) extreme FGT.

Figure 5 compares the breast density assessments of the 3 radiologists and the percentage of FGT present within the breast based on model predictions on the test set. The Pearson coefficient was 0.80 for ground-truth FGT percentages versus radiologist assessments and 0.75 for model-predicted FGT percentages versus radiologist assessments.

## Discussion

In our study, we developed two CNNs to accurately segment breast, FGT, and blood vessels using pre-contrast fat-suppressed T1-weighted MRI volumes. We created a radiologist-reviewed dataset using well-defined criteria to train these models. The dataset, code, and models were made publicly available.

Our deep learning method achieved a segmentation performance similar to or greater than previous methods. There have been a variety of studies that have used non-deep learning techniques to segment the breast and FGT. The most common methods are atlas-based fuzzy C-means methods and level-set-based methods, achieving DSC values ranging from 0.61 to 0.97 for breast segmentation and DSC values ranging from 0.80 to 0.84 for FGT segmentation^26–34^. There are also many studies that utilized deep learning, also employing either 2D or 3D U-Net models or similarly designed CNNs. They found DSC values ranging from 0.86 to 0.94 for breast segmentation and DSC values ranging from 0.83 to 0.92 for FGT segmentation^12–16^. Past works have compared a single U-net versus consecutive U-nets, similar to our work, for segmentation of breast and FGT, with Dalmış et al demonstrating superior performance with a single U-net^12^. However, our work includes blood vessel segmentation which is rarer tissue within the breast to segment, which may emphasize the need for consecutive U-nets to provide a breast mask for context.

Our analysis which compared breast density assessments between radiologists showed that there can be a large amount of variability between density assessments, with Cohen’s kappa values of 0.38, 0.65, and 0.43. This further illustrates the need for a standardized method of breast density assessment. FGT percentage is the calculation that can be employed from our model’s predictions of breast and FGT. When comparing FGT percentages and radiologist assessments, we demonstrated Pearson’s correlation coefficients of 0.80 and 0.75 for ground-truth and model-predicted FGT percentages, respectively. These indicate that i) FGT percentages are moderately correlated with breast density assessments, as expected, and ii) our model is able to retain a very similar level of correlation.

Although we achieved similar DSC values for breast and FGT segmentation to previous studies, a unique distinction of our study is the addition of blood vessel segmentations. Within the breast, there are other structures that can appear similar to FGT as they all appear similarly hyperintense on T1w fat-suppressed pre-contrast images. These hyperintense structures are mainly blood vessels and lymph nodes. Lymph nodes are most commonly present in the axillary region but intramammary lymph nodes can occur within the breast. When present, intramammary lymph nodes are usually small, few in number, and represent a very small proportion of the breast. As a result, we excluded them from FGT annotations and did not perform a separate annotation for them. In contrast, blood vessels are always present in the breast and can represent a sizeable proportion of the breast. In our annotations, out of all voxels that were labeled as either FGT or blood vessels, blood vessels accounted for 5.7% of the volume. As far as we are aware, previous studies that performed FGT segmentation did not account for blood vessels and therefore may be overestimating FGT by approximately 5.7% if blood vessels were included in FGT annotations.

The main limitation we faced in our study was the number of fully annotated MRI volumes. Despite using techniques such as intensity thresholding and 3D annotating tools, annotating a single MRI volume took approximately 8 hours. However, since we randomly selected patients, we believe that our dataset provides a heterogenous cohort for analysis with patients in a variety of scanners and with a variety of demographics. Furthermore, the size of our test set may have limited our comparison of models. There was no statistically significant difference between 3D models that included and did not include breast mask predictions, but such a difference was present when comparing 2D models. This is likely due to the small number of 3D volumes (n = 15) but many 2D slices (n = 2578).

## Conclusion

Our study performed accurate segmentation of breast, FGT, and blood vessels using two consecutive 3D U-Nets. Additionally, all code and data used are provided online for public use. Our deep learning method has the potential to objectively evaluate breast density in women to improve breast cancer risk assessments.

## Data Availability

The MRI studies used in this article are from the Duke Breast Cancer MRI dataset which is available online at 10.7937/TCIA.e3sv-re93, under “Data Access”, “Supplemental Segmentation”, “3D Breast and FGT MRI Segmentation Supplemental Data”. All code and trained models used in the study are publicly available at: https://github.com/mazurowski-lab/3D-Breast-FGT-and-Blood-Vessel-Segmentation.
